# Primary health care facility readiness to implement primary eye care in Nigeria: equipment, infrastructure, service delivery and health management information systems

**DOI:** 10.1186/s12913-021-07359-3

**Published:** 2021-12-20

**Authors:** Ada Aghaji, Helen E. D. Burchett, Ngozi Oguego, Shaffa Hameed, Clare Gilbert

**Affiliations:** 1 Department of Ophthalmology, College of Medicine, University of Nigeria, Enugu, Nigeria; 2 International Centre for Eye Health, London School of Hygiene & Tropical Medicine, London, UK; 3 Global Health and Development, Faculty of Public Health and Policy, London School of Hygiene & Tropical Medicine , London, UK; 4 Department of Ophthalmology, University of Nigeria Teaching Hospital, Enugu, Nigeria

**Keywords:** Primary eye care, Nigeria, Service delivery, Equipment, HMIS

## Abstract

**Background:**

Over two-thirds of Africans have no access to eye care services. To increase access, the World Health Organization (WHO) recommends integrating eye care into primary health care, and the WHO Africa region recently developed a package for primary eye care. However, there are limited data on the capacities needed for delivery, to guide policymakers and implementers on the feasibility of integration. The overall purpose of this study was to assess the technical capacity of the health system at primary level to deliver the WHO primary eye care package. Findings with respect to service delivery, equipment and health management information systems (HMIS) are presented in this paper.

**Methods:**

This was a mixed-methods, cross sectional feasibility study in Anambra State, Nigeria. Methods included a desk review of relevant Nigerian policies; a survey of 48 primary health facilities in six districts randomly selected using two stage sampling, and semi-structured interviews with six supervisors and nine purposively selected facility heads. Quantitative study tools included observational checklists and questionnaires. Survey data were analysed descriptively using STATA V.15.1 (Statcorp, Texas). Differences between health centres and health posts were analysed using the z-test statistic. Interview data were analysed using thematic analysis assisted by Open Code Software V.4.02.

**Results:**

There are enabling national health policies for eye care, but no policy specifically for primary eye care. 85% of facilities had no medication for eye conditions and one in eight had no vitamin A in stock. Eyecare was available in < 10% of the facilities. The services delivered focussed on maternal and child health, with low attendance by adults aged over 50 years with over 50% of facilities reporting ≤10 attendances per year per 1000 catchment population. No facility reported data on patients with eye conditions in their patient registers.

**Conclusion:**

A policy for primary eye care is needed which aligns with existing eye health policies. There are currently substantial capacity gaps in service delivery, equipment and data management which will need to be addressed if eye care is to be successfully integrated into primary care in Nigeria.

**Supplementary Information:**

The online version contains supplementary material available at 10.1186/s12913-021-07359-3.

## Background

In sub-Saharan Africa (SSA) an estimated 21.5 million adults are blind or visually impaired, 80% from avoidable causes. Uncorrected refractive error accounts for almost half of this visual loss and cataract for another third. In addition, over 100 million Africans have uncorrected presbyopia (the age related decline in near vision) which can easily be treated with spectacles [1]. In Africa, at least 40% of blindness in children is avoidable being due to corneal blindness from preventable conditions such as vitamin A deficiency, measles infection and neonatal conjunctivitis, or treatable conditions such as cataract [2].

Access to appropriate health care in SSA is challenging, and the Healthcare Access and Quality Index (HAQ), a measure of personal healthcare access and quality (range 0–100), is estimated to be only 19.6 in Africa compared with 54.4 globally [3]. Furthermore, the majority (approximately 70%) of people in Africa do not have access to eye care [4] as most eye care services are in secondary and tertiary facilities in urban areas [5], reducing access by rural populations. This is significant because inequitable access to eye health services is responsible for the majority of blindness and visual impairment [6]. Primary health care (PHC) services, on the other hand, are widely accessible to the majority of the population in urban and rural areas, and the World Health Organization’s (WHO) Global Action Plan (WHO GAP) 2014–2019 recommends that eye care becomes an integral component of PHC and health systems development [7]. The recent WHO World Report on Vision also recommends primary health care systems as the vehicle to deliver “integrated people-centred eye care.” [8].

In Nigeria, about 4.25 million people are blind or visually impaired and over 84% of the causes of vision loss could have been prevented or treated [9, 10]. Access to eye care is a major reason why people remain blind in Nigeria [11]. We anticipate that integrating eye care into PHC in Nigeria would increase access to eye care services. Primary level facilities in Nigeria consist of health centres and health posts. Health centres are larger, better equipped 24-h facilities that manage deliveries, while health posts are less well equipped and staffed and are only open for fixed hours during the day. Health centres are staffed by nurse/midwives (NMWs), community health workers (community health officers (CHO), community health extension workers (CHEW), junior community health extension workers ((J)CHEW) and sometimes doctors. Each health post is manned by a (J)CHEW [12]. PHC in Nigeria is governed centrally by the National Primary Health Care Development Agency (NPHCDA) and more recently at state level by Primary Health Care Development Agencies [13].

The WHO Africa region recently launched a package of interventions to integrate eye care into PHC in SSA (the WHO AFRO PEC package) which has been pilot tested in Kenya and Rwanda [14]. However, despite growing support for integration of eye care at PHC level, there are limited data on the capacities needed for delivery, to guide policy makers and implementers on the feasibility of integration [15]. In addition, indicators for monitoring the WHO AFRO PEC package have not yet been published. However, the key eye health indicators for the Africa region (2017) include whether primary eye care (PEC) is part of PHC, the number of patient consultations for eye diseases and the number of relevant eye medications on the essential drug list [16].

Human resources for health (HRH), governance and health financing are crucial for health care delivery [17]. Health systems are composed of interconnected elements which must work together to be effective, as adjustments in one area may affect others [18]. The other elements of the health system i.e., service delivery, health management information systems, and equipment, technology, and consumables, also have a key role to play to achieve improved health outcomes. This article reports a component of a mixed methods study to determine the technical feasibility and the health system capacities required to deliver the WHO AFRO PEC package in PHC facilities in Anambra State, Nigeria.

## Methods

Methods leading up to the facility survey included a literature review to identify a relevant theoretical feasibility framework, a literature review of PEC in sub-Saharan Africa, a Delphi exercise to finalize statements on the technical feasibility and capacities needed to deliver the PEC package, and the development of a number of study instruments based on the agreed statements [19, 20]. In this paper we report the findings of a policy document review and facility survey in relation to service delivery, health management information systems (HMIS) and equipment, technology and consumables.

### Desk review of policy documents

The desk review included a range of policy documents of relevance to the delivery of PHC and eye care in Nigeria (Additional file 1). Statements on service delivery, equipment, consumables, infrastructure and the data collected for HMIS which would support the WHO AFRO PEC package were extracted and mapped onto the WHO health systems framework [21].

### Study location

The PHC facility survey was conducted in Anambra state in south-eastern Nigeria which has a population of 5.53 million [22]. 75.1% of the population aged ≥6 years are literate [23] and 11.3% are poor [24]. There are two tertiary hospitals, 35 secondary hospitals, and 347 PHC facilities comprising 235 health centres and 112 health posts.

### Facility survey and interviews with supervisors and facility heads

Details of how facilities were selected for the study are described in detail in a protocol paper [19]. In brief, 48 PHC facilities in six districts were selected using two stage, stratified random sampling, ensuring a proportionate mix of health centres and health posts, in rural, urban or semi urban locations.

Observational checklists were used to assess infrastructure, equipment, drugs and consumables and data recording systems, including the number of patients who attend the facility overall and by age group. Structured questionnaires were administered to facility heads (who comprised a range of different cadres) about the services provided, and referral activities and mechanisms. Nine facility heads were purposively selected for in-depth interviews based on an interim analysis to identify the highest and lowest scoring facilities in terms of patient attendances /1000 population, the health workforce and regularity of supervision. These were stratified by location (urban, rural or semi urban) and type of facility (PHC or health post). Six health centres and three health posts were selected.

Semi-structured interviews were conducted with district supervisors for health in the six selected districts. Topic guides were used to explore the challenges they encounter in delivering PHC and their views on the feasibility of delivering PEC. All interviews were conducted in English by the principal investigator (AA) and were recorded after informed consent had been obtained.

### Data management

Data were recorded on paper forms and entered into separate Microsoft Access® databases for the questionnaire and checklists and were transferred into STATA V,15.1 (Statcorp, Texas) using STATransfer for analysis. Frequency tables were generated from the data. Simple descriptive analyses were performed e.g., the proportion of facilities visited with space for visual acuity assessment. Differences in quantitative variables between health posts and health centres were explored using the z-test statistic. Tests of significance were set at the 95% level. The interview recordings were transcribed verbatim, checked for accuracy and coded by AA. The WHO health systems building blocks were used as the framework for analysis after familiarisation of the data by re-reading, indexing, charting, mapping and interpretation. Open Code Software V. 4.02 was used to assist analysis.

### Ethics

Ethical approval was obtained from the ethics review boards of the Federal Ministry of Health, Nigeria, the University of Nigeria Teaching Hospital and the London School of Hygiene & Tropical Medicine. Permission to collect data from the state Ministry of Health and district departments of health was obtained. All participants gave written informed consent including to audio record interviews and use of anonymous quotes where appropriate. The following steps were taken to ensure confidentiality; no names were collected for any component of the study and each facility and participant was allocated a unique code. Supervisor codes did not include the district.

## Results

### Policy document review

Nigeria has no specific policy for PEC. However, recent national health policies support eye care delivery at the PHC level. For example, the National Health Policy 2016 recommends building capacity for eye care services delivery at all levels, including PHC level [25].

In addition, as part of the minimum package for prevention and control of non-communicable diseases, the NPHCDA recommends the provision of primary eye care to reduce preventable blindness which contributes to increased morbidity and mortality [13].

The recent National Strategic Health Development Plan (NSHDP II) 2018–2022 includes eye care as part of the non-communicable disease package at PHC level and recommends scaling up eye care services at all levels, particularly at PHC level. The NSHDP II also recommends the assessment of the prevalence and causes of vision loss to generate more current data and track trends over time. In terms of medication, the NSHDP II recommends that Erythromycin ointment for ocular prophylaxis of the new-born be included in the package for new-born health at all level, including PHCs [26].

Chloramphenicol eye drops and ointment, and Chlortetracycline ointment are the only eye medicines on the Essential Drugs list to be available at primary health centres [27]. The NPHCDA Minimum standards for PHC in Nigeria 2015 recommends that Snellen’s charts and pen torches should be available in health centres but not in health posts [12].

The National Health Act (2014), mandates the establishment of the Basic Health Care Provision Fund: of which 20% should be used for essential drugs, vaccines and consumables15% of the fund be used to provide and maintain facilities, equipment and transport for eligible PHC facilities [28]. An additional file shows the relevant sections of the policy documents in more detail. (See Additional file 1).

### Sample characteristics of survey

Surveys including observational checklists were conducted in 48 facilities: 33 primary health care centres and 15 health posts. Data were obtained from all heads of facilities. Four of the 48 (J)CHEWs were unavailable (on study or maternity leave). Interviews were conducted with nine facility heads and six district supervisors for health (Table 1).

**Table 1:** Sociodemographic characteristics of the interviewees

Identification code	Age group (years)	Sex	Qualification	Facility type	Location
**Supervisors for health**					
SUP/1	51–60	Male	Medical Doctor	^a^see below	
SUP/2	51–60	Female	Community Health Officers		
SUP/3	51–60	Male	Medical Doctor		
SUP/4	51–60	Male	Medical Doctor		
SUP/5	51–60	Female	Community Health Officers		
SUP/6	51–60	Male	Medical Doctor		
**Heads of facility**					
HoF/HP/U/1	51–60	Female	Com. Health Extension Worker	Health Post	Urban
HoF/PHC/U/2	31–40	Female	Nurse midwife	Health Centre	Urban
HoF/PHC/U/3	31–40	Female	Community Health Officer	Health Centre	Urban
HoF/PHC/SU/4	51–60	Female	Nurse midwife	Health Centre	Semi urban
HoF/PHC/SU/5	41–50	Female	Nurse midwife	Health Centre	Semi urban
HoF/HP/SU/6	41–50	Female	Community Health Officers	Health Post	Semi urban
HoF/HP/R/7	31–40	Female	Com. Health Extension Worker	Health Post	Rural
HoF/PHC/R/8	31–40	Female	Community Health Officers	Health Centre	Rural
HoF/PHC/R/9	51–60	Female	Nurse midwife	Health Centre	Rural

^a^Location not documented to maintain confidentiality. Facility type is not applicable here.

### Findings from the facility survey and interviews

#### Infrastructure, equipment and consumables

Over 90% of facilities had space to measure visual acuity (6 m) but less than 5% had a visual acuity chart (Table 2). Less than a third of the facilities had a functioning torch and less than half had secure (i.e., lockable) storage for medication or a functioning refrigerator. It seems that staff in facilities are responsible for repairing and, in some cases, procuring equipment, or staff charge a small fee to cover the cost of replacement, as mentioned by two facility heads:

**Table 2:** Infrastructure, equipment and consumables in 48 primary health care facilities

	Health centre		Health post		Total		Difference	
	N	%	N	%	N	%	%	*p* value
**Infrastructure and equipment**								
Space to measure visual acuity	32	97.0	13	86.7	45	93.8	10.3	0.2
Visual acuity chart	1	3.0	1	6.7	2	4.2	NA	
Torch available	17	51.5	5	33.3	22	45.8	18.2	0.5
Torch available and functioning	11	33.3	3	20	14	29.2	13.3	0.7
Space for counselling	27	81.2	7	46.7	34	70.8	35.1	0.6
Secure drug storage	13	39.4	9	60	22	45.9	20.6	0.3
Functioning refrigerator	18	54.6	4	26.7	22	45.8	27.9	0.3
**Medication of relevance to eye care**								
Vitamin A 200,000 IU in stock	29	87.9	10	66.7	39	81.5	21.2	0.1
Vitamin A 100,000 IU in stock	25	75.8	10	66.7	35	72.9	0.9	0.6
No vitamin A in stock	4	12.1	2	13.3	6	12.5	1.2	1.0
Access to measles vaccine	30	90.9	14	93.3	44	91.7	0.3	0.8
Tetanus toxoid	22	66.7	4	26.7	26	54.2	40	0.1
Injectable antibiotics	28	84.9	6	40	34	70.8	44.9	0.02
**Medication for eye conditions**								
Antibiotic eye drops	4	12.1	0	0	4	8.3	12.1	–
Antibiotic eye ointment	5	15.2	0	0	5	10.4	15.2	–
Anti-allergy eye drops	0	0	0	0	0	0	0	–
**Consumables for eye care**								
Saline	21	63.6	5	33.3	26	54.2	30.3	0.2
Cotton wool	29	87.9	12	80	41	85.4	7.9	0.5
Gauze	24	73.7	6	42.8	30	63.8	29.9	0.2
Plasters	23	69.7	3	20	26	54.2	49.7	0.09
Bandages	8	24.2	2	13.3	10	20.8	10.9	0.7

*But in case we don’t have a sphyg [sphygmomanometer] or it gets spoilt, you have to go and buy (it) because you cannot be waiting for them until they provide a syphg which you use on a daily basis. You have to go and buy to make sure that the work continues*. HoF/PHC/SU/5

*Here’s what we do….. this sphyg that is functioning, we collect 50 Naira [£0.12] when someone comes for the services, so if it gets spoilt, we will use the N50 collected and replace it, so that when people come this service will still be available*. HoF/PHC/U/2

The majority (81.5%) of facilities were observed to have vitamin A 200,000 IU in stock but almost 30% did not have the lower dose (100,000 IU) for younger children (Table 2). Six facilities (12.5%) had no vitamin A. Over 90% of facilities had access to measles vaccine (i.e., either in stock or provided on measles immunization days) but over 85% of health centre, and 100% of health posts, lacked medication for eye conditions.

Facility heads reported that drugs are obtained from multiple sources and that staff also purchase drugs:

*SOML (Saving One Million Lives, a federal government initiative to increase access to maternal and childcare services at PHC level) brings [the drugs], then the state, then the local government commission. When they bring [the drugs] we go and pay for them…..then we buy ours and add to them because they do not give us all the drugs we need.* HoF/PHC/SU/4

*We used to buy our medications but recently SOML started bringing medications. There are some that the (district) supervisor brings (*HoF/HP/R/7

There may also be challenges with the quality of the drugs supplied, as explained by a facility head:

*What upsets us is that the drugs they give us….. are inferior drugs, they bring us inferior products. They are difficult for us to dispose of. When you give it to a patient, they’ll say “what kind of drug is this that looks like imitation?” But if we are going to buy the drugs ourselves, we know the best companies to go to for [drugs] that work. But here they supply us with drugs that we know nothing about and then they tell you how much you’ll pay for them*. HoF/HP/SU/6

A district health supervisor had a different view and explained some of the challenges they face in how drugs are managed in facilities:

*There was a drug revolving fund, but we found out that some of the health workers will keep the drugs and will be selling their own drugs….. A lot of our drugs, … are good drugs, but were on the expensive side. Even among the supervisors we have discussed about that. We say, “these things are expensive.” If you give them to these officers in charge [heads of facility] they will just pack them there and use their own money to buy their own. At the end of the day, they will tell you, “this thing has expired”. So, you find out that when you are talking about this drug revolving fund, you really need to find out which ones the people can afford.* SUP/1

### Service delivery

#### Scope of services delivered and patient load

Overall, the mean number of patient visits per 1000 catchment population per year was similar for health centres (mean 316 ± 257) and health posts (301 ± 350) (*p* = 0.9). Antenatal care was available in over 90% of the facilities, and 97% of health centres and 26.7% of health posts provided services for deliveries (Table 3). Measles immunization was provided by all facilities and 93.8% provided vitamin A supplementation. Crede’s ocular prophylaxis to prevent neonatal conjunctivitis was not delivered in any facility, and eye care services were available in less than 10% of facilities. Less than half of the facilities provided services for the elderly, and over half (52.1%) of the facilities saw ≤10 elderly persons/1000 catchment population in the year 2017. Blood sugar testing was available in a little over half of the facilities.

**Table 3:** Service delivery in primary health care facilities

	Health centre		Health post		Total		Difference	
Services provided	N	%	N	%	N	%	%	*P* value
*Maternal health services*								
Antenatal care	33	100.0	11	73.3	44	91.7	26.7	0.002
Deliveries	32	97.0	4	26.7	36	75.0	70.3	0
*Child health services*								
Measles vaccination	33	100.0	15	100.0	48	100.0	0.0	
Vitamin A supplements	33	100.0	12	80.0	45	93.8	20.0	0.008
Credes prophylaxis	0	0.0	0	0.0	0	0.0	0.0	
*General healthcare services*								
Diabetic care	21	63.6	6	40.0	27	56.3	23.6	0.3
Elderly care	16	48.5	5	33.3	21	43.8	15.2	0.6
Eye care	3	9.1	1	6.7	4	8.3	2.4	0.9
Blood pressure measurement	33	100.0	15	100.0	48	100.0	0.0	
Providing 24-h services	29	87.9	3	20.0	32	66.7	67.9	0.004
**Total patient visits per year (2017)**								
≤ 1000	7	21.2	7	46.7	14	29.1	25.5	0.3
> 1000 to ≤2000	15	45.5	6	40	21	43.8	5.5	0.8
> 2000 to ≤4000	8	24.2	2	13.3	10	20.8	10.9	
> 4000	3	9.1	0	0	3	6.3	9.1	NA
Mean number of patient visits	2201 ± 1421		1136 ± 753		1745 ± 1310		1065	0.0015
**Patients visits /1000 catchment population per year (2017)**								
≤ 250	16	48.5	8	53.5	24	50.0	5.0	0.8
> 250- to ≤500	10	30.3	3	20.0	13	27.1	10.3	0.7
> 500	7	21.2	4	26.7	11	22.9	5.5	0.8
Mean number of patient visits	316 ± 257		301 ± 350		312 ± 283		15	0.9
**Patients aged over 50 years visits /1000 catchment population year (2017)**								
≤ 10	17	51.4	8	53.3	25	52.1	1.9	0.9
> 10 to ≤20	6	18.2	1	6.7	7	14.6	11.5	0.8
> 20 to ≤50	5	15.2	3	20.0	8	16.7	4.8	0.8
> 50	5	15.2	3	20.0	8	16.7	4.8	0.8
Mean number of patient visits	21 ± 27		16 ± 19		19.5 ± 25.1		5	0.47

A head of facility and a supervisor realised the limitations of the scope of services they provide, which do not address the health needs of a large proportion of the population, including for eye care.

*That is the problem with the system - they always focus on children under 5, pregnant women, women of childbearing age, that is mostly their work. …..there is no place for the elderly, the handicapped - here is spacious enough that we can put it in.* HoF/PHC/U/3

*In fact, thinking about it, I wonder why no one thought of including it (eye care) in primary health care.* SUP/6

#### Eye care services

Staff were asked about their ability to deliver eye care services. Although most (84.1%) were confident in their ability to identify eye conditions, less than a third were confident in their ability to manage eye conditions and two thirds (63.4%) had not referred a patient with an eye condition in the previous month. In only a few facilities was someone available to remove a foreign body from the eye (27.3%) or who could irrigate the eyes (29.5%) for chemical injuries.

An unexpected finding that emerged during interviews was that eye care is provided in some facilities by visiting eye care professionals.

*Sometimes, there are people that come here to treat the eyes….they tell us they are coming….. we do an announcement, tell the community that people who will treat the eyes are coming, that’s what we do.* HoF/HP/R/7

*Let me give you an instance. Last year in June… there was a group of people who came…. They wanted to provide eye care services. They came from [name of town]. They wanted to provide eye care services in every facility in this district.* HoF/PHC/U2

One of the supervisors who had an eye problem herself wished to consult the eye care provider but could not because there were too many people.

*Like the other day they did this free medical clinic, a lot of people were asking… I went into the hall where they were seeing people that have eye problems, there were a lot of people. Myself, I wanted to see the [eye] doctor [for reading glasses], I was not able to see the [eye] doctor.* HoF/PHC/R/9

Supervisors generally granted permission for eye care providers to visit facilities and deliver services as they realized the importance of eye care:

*We actually need this eye health thing. It is very, very important. We don’t just allow anyone to enter into the community [to provide eye care]. SUP*/1

*They [the eye care providers from the named town] discussed with the supervisor - he approved. They gave us letters and issued dates [for their visit].* HoF/PHC/U2

However, it is unclear whether these service providers were regulated, as it appears that medication was initially provided at no cost but after some time the providers started to charge for medication, as reported by this head of facility:

*When they started initially…. they said that it was free… but later they started taking money. So, after announcing that the treatment is free and they come and start charging for medication, the patients are not happy. They were coming out in large numbers initially, but when money became involved, they stopped coming out in their numbers. Only those who had serious eye problems started coming*. HoF/HP/R/7

#### Health service data collection

All the facilities used paper-based data collection methods. Almost all kept patient registers and about two thirds had drug inventories. Inventories for consumables were generally lacking (Table 4). On observation of patient registers, no patients with eye conditions were recorded. This finding was explained by a head of facility, which was corroborated by a supervisor:

*We don’t treat eyes, so we don’t record them.* HoF/HP/U/1

*They don’t record [patients with eye conditions]. They refer immediately they see [them] because there is nothing (they can do)… but before they refer, at least they must have done some primary care, like asking questions, but because it is not in their daily register, they don’t record the data.* SUP/5

**Table 4:** Register and inventories and referral mechanisms in health centres and health posts

	Health centre		Health post		Total		Difference	
	N	%	N	%	N	%	%	*p* value
**Registers / inventories**								
Patient register	32	97.0	15	100	47	97.9	3	0.5
Medication inventory	23	69.7	9	60	32	66.7	9.7	0.6
Facility activities register	22	66.7	9	60	31	64.6	6.7	0.7
Consumable inventory	2	6.1	0	0	2	4.2	6.1	–
**Referral mechanisms**								
Referral register	9	27.3	0	0	9	18.8	27.3	–
Referral slips	9	27.3	0	0	9	18.8	27.3	–

In addition to the registers, staff are also required to collect data in disease or programme specific registers, which entails a lot of work in documenting and compiling the data for monthly reports. One head of facility said that she had employed a volunteer to help with data management as it was so time consuming.

*We have general attendance registers, out-patient department register, in-patient register, delivery register, we have immunisation register, we have growth monitoring register. All those registers… as they (patients) come, we register. At the end of the month, you summarize everything in the summary booklet and then submit (to the district) ….they have to submit it to the State.* HoF/PHC/U/3

*I’ve been working alone…. (I need a volunteer) to help me, even if it’s just to fill the registers… so that I can manage the treatment and other things.* HoF/PHC/R/8

Other facility heads described the diseases they are required to report to the district.

*We submit data on antenatal care, immunisation, total number of patients that come, notifiable diseases like measles, AFP (acute flaccid paralysis) if you have any cases, cough with acid fast bacilli in sputum, tetanus in adults and neonatal tetanus.* HoF/PHC/R/8

One of the supervisors thought that if the number of patients attending with eye conditions were also to be reported this would either require a separate column in the general patient register, or a separate register.

*So, you create a column for each condition; so that anyone that you see on daily basis - that will be noted and at the end of the month it will be compiled because there are a lot of registers with us. It has to be inclusive; the eye column should be included in the registers so as to make easy flow of the registers. Getting separate registers should be done. But if the Ministry of Health welcomes that in a primary health care setting, it should be included.* SUP/2

Most of the facilities (73%) had designated referral centres. 43% of heads of facilities confirmed that their designated referral centres had eye clinics while 28% were unsure. Less than 20% of facilities used referral slips, and it seems that health workers refer patients by word of mouth, as mentioned by two facility heads:

*We don’t have referral slips in this facility.* HoF/PHC/U/2

*We refer by [word of] mouth.* HoF/PHC/U/3

## Discussion

### Policy gaps

Recent national policies show that there is an enabling policy environment for eye care at PHC level, but there is not a specific policy for PEC. Currently no data are collected or reported on eye conditions, as they are not one of the indicators in the HMIS at primary level. However, this study shows that when eye care is provided, patients attend in large numbers, demonstrating an unmet need. The failure to implement the policies on eye care at primary level may have multiple causes, including lack of data which demonstrates demand, the inevitable lag between policy development and implementation, and/or lack of advocacy or leadership. The development of a specific PEC policy, which brings together and adds to existing policies (e.g., for new-born care, non-communicable diseases and care of the elderly) with indicators may help to drive the delivery of eye care at PHC level. If the uptake is high, eye data will help to assess the magnitude of eye conditions and whether and how they change over time, as recommended by the National Strategic Health Development Plan 2018–2022.

A policy gap exists in the new-born policy recommendation that erythromycin antibiotics be instilled in the eyes of every new-born to prevent neonatal conjunctivitis as the current essential drugs list for PHC facilities does not include erythromycin. There is need for eye health stakeholders to advocate for the inclusion of erythromycin at PHC level in the next revision of the essential drugs list to enable implementation of this policy.

It appears that eye medications should not be stocked by health posts, as they are only included in the essential drug list for health centres. The implication is that eye care will be delivered at higher level than health posts or there could be a policy change to accommodate this.

### Survey findings

The key capacity gaps in relation to delivering PEC identified in the facility survey include a dearth of eye care equipment and drugs, patients with eye conditions are not being recorded in the patient registers and that a low number of elderly persons visit PHC facilities.

### Infrastructure, equipment and drugs

There was no significant difference between health centres and health posts in terms of availability of equipment, infrastructure and consumables for PEC. Visual acuity charts were not available in the majority of facilities and less than a third had functioning torches. However, the majority had space for visual acuity assessment. This is in contrast to studies in East Africa where visual acuity charts were more likely to be available in facilities where staff had undergone pre-service training in PEC, and where supervisors had also been trained in PEC [29].

Maternal and child health is one of the priority functions of PHC. Therefore, vitamin A, which plays an important role in reducing under 5 mortality and morbidity, including blindness, should always be in stock in all PHC facilities. In this study vitamin A was not available in a minority of facilities, in contrast to another study in Anambra state, where vitamin A was only available in three quarters of PHC facilities [30]. Low vitamin A supplementation coverage has been shown to be associated with corneal blindness in children in Nigeria [31]. No facilities had topical medication for allergic conjunctivitis which is included in the WHO AFRO PEC essential drug list [32] but is not on the drug list for PHC facilities.

Antibiotic eye medications (chloramphenicol eye drops and tetracycline ointment) are included in the Essential Medications List for PHCs in Anambra State but very few facilities had these in stock. This is likely to reflect the low demand, as until recently, eye care was not included in policies at PHC level. Erythromycin ointment has recently been recommended for ocular prophylaxis in new-borns [26], but this is not included in the essential drug list for PHC facilities in Nigeria [27], which needs to be addressed. For drugs of relevance to eye care to be available in PHC facilities they need to be included in the country’s essential drug list for PHC, but this is not the case in many low-income countries [33], and their inclusion will require advocacy and multilevel coordination.

In this study staff in facilities reported that the drugs provided were insufficient in quantity, came from multiple sources, were too expensive or not acceptable to patients. To address these limitations staff bought drugs themselves. While it is commendable that staff used their initiative to address this critical gap, it suggests weak regulation of drug supplies. Whether the same would happen with topical eye medication remains to be seen. Most health centres had injectable antibiotics in stock, an essential drug for health centres but not health posts [27]. Staff also reported dissatisfaction with the quality of the drugs supplied. Although it is possible that some products may have been sub-standard, there is no evidence of any quality assurance to support or refute this claim. The Bamako initiative was launched by UNICEF in 1987 to improve the quality of PHC and increase access to medications at PHC level [34]. While there are several reports on the availability or non-availability of drugs at PHC facilities, there is limited data on the quality of drugs supplied [30, 35]. Policy makers should place additional emphasis on the quality of drugs procured.

### Service delivery

The majority of services provided in PHC facilities focussed on maternal and child health which was reflected in the age of those attending the services i.e., very few over the age of 50 years. This is similar to a study in Enugu, Nigeria where the focus was on immunisation, treatment of minor ailments and maternal and child health services, and where less than 7% of people using PHC services were above 47 years of age [36]. In another study in North central Nigeria, almost 40% of patients attending health centres were infants aged 0–9 months [37]. These findings present a challenge to the delivery of PEC as the prevalence of visual impairment increases with age [38]. Assuming 8% of the population are aged 50 years and above and 4% are blind or visually impaired there will be approximately 64 affected individuals of this age group in the catchment population of a health centre (20,000). In addition, there will be a far larger number of adults with presbyopia, which affects most people over the age of 50 years, as well as children and adults with other non-visually impairing conditions. In order to increase access by individuals with eye conditions of all ages once PEC is in place, particularly by adults and the elderly, extensive health promotion and demand creation may be required.

Crede’s prophylaxis to prevent neonatal conjunctivitis, a documented cause of blindness in children in southeast Nigeria [39], was not practised in any facility despite an enabling policy. Strategies to instate prophylaxis could include training health workers including supervisors, educating mothers and providing appropriate medication.

An unexpected finding was that some PHCs are visited by eye care professionals according to a pre-determined schedule. These services were attended by a large number of people when medication was provided at no cost. Once costs were introduced, only those with serious eye conditions continued to attend. These findings reflect the large unmet need for eye care in the community and suggest that people with eye conditions are likely to seek care if demand is created, they have confidence in the provider, and affordable services are provided. Many staff were confident in their ability to identify people with eye conditions but due to lack of in-service training they lacked confidence in how to manage them.

Almost half the facilities did not provide blood sugar testing despite being included in the minimum package for the prevention and control of non- communicable diseases in PHC facilities in Nigeria [13]. The implications are that diabetes and diabetic retinopathy may go undetected.

### Health service data collection

The majority of facilities had patient registers, but up to a third lacked inventories for medication and even fewer had an inventory for consumables. None of the facilities recorded details of patients with eye conditions in their patient registers as they could not treat them. Staff could not, therefore generate or report data on the number of eye patients seen, despite this being an indicator for eye care for the African region. Similar findings have been reported from Tanzania [40] and South Africa where less than 5% of facilities collected eye health indicators [41]. However, in Rwanda, where PEC is integrated into national policies and programmes, eye health data are collected and reported at all levels to monitor progress and identify gaps that need to be addressed in secondary and tertiary eye care [42]. The lack of data from PHC level on eye care in Nigeria will impede public health decision making for PEC, as this requires robust, timely data [43]. A challenge in collecting data on eye care in PHC facilities is that this will add to the administrative workload of PHC staff who already have to compile and report data for numerous programmes and notifiable diseases.

In this study less than a fifth of facilities had referral slips or referral registers, and referrals are made orally, as had been found in other studies [44]. The lack of organised referral systems in PHC facilities, including feedback from higher level facilities, leads to lack of continuity of patient information which is likely to compromise care, particularly for patients requiring long term management [44]. Less than half of the facility heads knew whether their designated referral centre provided eye care services and almost a third were unsure. Many eye conditions which could be detected at PHC level e.g., refractive error and cataract, need to be treated at referral centres. To facilitate referrals, PHC facilities will need to be mapped to the nearest government eye care provider.

### Strengths and limitations

This is the first study to assess the technical capacity of PHC to deliver the WHO AFRO PEC package. A potential limitation of this study is that the sample of PHC facilities was not adequately powered to detect statistically significant differences between health centres and health posts, but every effort was made to make the sample as representative as possible in terms of health centre to health post ratios and the location of facilities. Another limitation is that the data collected focussed on facility level and the views of district level supervisors and did not include community perspectives or an assessment of eye care facilities for referrals, nor upward transmission of facility level data.

## Conclusions

The key gaps for delivering the WHO AFRO PEC package identified in this component of the study include a dearth of equipment and drugs for eye care, the non-inclusion of patients with eye conditions in facility registers, weak referral systems and low numbers of older adults and the elderly who visit PHC facilities. Many of these issues could be addressed by modifying and implementing existing policies, sustained advocacy, and leadership, with health promotion and community mobilization to increase demand.

Further research is needed to assess the need for and acceptability of PEC for people of all ages in communities in Anambra state and to assess whether secondary and tertiary level eye care facilities have the capacity to manage referrals. The findings will complement this PHC facility survey, providing contextually relevant data and information for decision makers.

## Supplementary Information

**Additional file 1.** National Health Policy Documents Reviewed. Health policies that support the implementation of PEC in Nigeria with regard to service delivery, HMIS, infrastructure, equipment and consumables.

**Additional file 2.** Framework for semi structured interviews.

## Data Availability

The dataset generated and analysed for this study are available from the corresponding author on reasonable request.

## Abbreviations

AFRO: Africa region
CHO: Community health officer
GAP: Global action plan
HMIS: Health management information system
HOF: Head of facility
HP: Health post
HRH: Human resources for health
(J)CHEW: (Junior) community health worker
LSHTM: London School of Hygiene & Tropical Medicine
NHREC: National Health Research Ethics Committee
NMW: Nurse mid wife
NPHCDA: National Primary Health Development Agency
NSHDP: National Strategic Health Development Plan
PEC: Primary eye care
PHC: Primary health care
SOML: Saving one million lives
SSA: Sub Saharan Africa
SUP: Supervisor
WHO: World Health Organization
