# Supplementary material for: Primary health care facility readiness to implement primary eye care in Nigeria: equipment, infrastructure, service delivery and health management information systems
Source: BMC Health Serv Res. 2021 Dec 20;21:1360. doi: 10.1186/s12913-021-07359-3 (PMC8690487; doi:10.1186/s12913-021-07359-3)
Supplement: Supplementary file 2 — Additional file 2. Framework for semi structured interviews. [file 12913_2021_7359_MOESM2_ESM.docx]

**Topic Guide: Head of Facility**

**Aim: To assess the perceptions/experiences of facility heads about PEC implementation in the facility.**

**Introduction**

How long have you worked here?

What is your main role in this facility?

Next, I would like to ask you some questions about your role as the Head of this Facility.

1. **Leadership and Governance.**

- I understand that you supervise Health Promotion Activities in this community. What has your experience been like?
  - *Probe: What groups are targeted? e.g. new-borns, mothers and their young children, the elderly.*
  - *Probe: What health promotion activities are supervised.*
  - *Probe : What have been your challenges and opportunities. Ask for personal examples.*
  - *Probe for how eye health activities are supervised.*

1. **Human Resources for Eye Health**

- As a member of this community, can you tell me who people usually consult first when they have a health problem?
  - *What about people with eye conditions – who do they usually consult first? What do you think about that?*
  - *What would you feel about working with these groups to encourage them to refer eye patients to this facility?*
- Could you describe what happens when a patient with eye problems comes to this facility? Give an example of when it happened.
  - *Probe for what knowledge and skills staff have to handle eye cases.*
  - *Probe for how confident (s)he is in supervising PEC and what resources (s)he may need to effectively carry out PEC supervision-training, time, supervision.*
  - *Probe for what in service training is available for health workers and whether any is available for PEC.*

1. **Service Delivery**

- I understand that the facility sometimes produces key messages for health promotion. Can you explain the process of how you do this? Give examples from your personal experience.
  - *What do you think about incorporating key messages for promoting eye health?*
  - *What has been your experience in demand creation for a new intervention? Give an example. e.g. Insecticide treated mosquito nets.*
- Are any NGOs supporting services in this facility or in the community?
  - *Probe: what do they support?*
  - *How effective has their support been?*
  - *In what way can these NGOs support eye care?*

1. **Equipment Technology Consumables.**

- Do you face any difficulties in sourcing equipment for this facility?
  - *Probe for how one gets equipment fixed when it breaks down.*
- Based on your experience, do you think there will be any problems sourcing eye care equipment in this facility? *(Snellen distance visual acuity chart; near visual acuity chart, torches and batteries).*
- Based on your experience, do you think there will be any problems sourcing consummables in this facility?? *(saline, cotton wool, gauze, plaster)*
- How would easy will it be to stock eye medications in this facility.
  - *Probe for how easy it will be to stock eye medications, what the cost of medications are, what the demand for eye medications is like.*
- How is eye care medication dispensed to patients in this facility with eye conditions who need them?
  - *Probe for who prescribes the medication. Probe for how much the drugs cost.*
  - *Probe: What happens when stock outs occur? Please give an example of when this occurred.*
- Who in the facility is responsible for maintaining stocks of medication?
  - *Probe for what training if any they have had in this?*

1. **Health Management Information Systems**

I now would like to ask you some questions about the information you record in this facility.

- Please can you explain to me who is responsible for completing the patient register?
- *Probe: How is patient attendance documented?*
- *Probe: If eye conditions have not been registered in this facility (from Quant tool), why is this the case?*
- What is the process for referring patients to referral centres?
  - *Probe for how compliant patients are.*
  - *Probe for assisted referrals-phone calls or transportation.*
  - *Probe in what way if any feedback is given from the referral centre.*
- In what way are staff shifts managed*?*
  - *Probe: Is this a 24 hour facility?*
- Probe: In what way do staff manage an inventory for drugs and consumables?
  - *Probe for record keeping.*
  - *Probe whether staff have received training for inventory management.*
- I understand that you collate data and send to the district. Is eye health data included?
- *Probe for who determines what data is sent to the district supervisor.*

Closing.

- How do you think demand for eye care in the community can be created?
- Some Primary Health Facilities in other states deliver PEC in their facilities. What is your opinion about that? Do you think it can work in your facility? Why do you say so?
- Name 3 of the most important things that will be necessary to make PEC work successfully in your facility?
- Finally. Is there anything left out or something you would like to mention regarding primary eye care in this facility?
- If you think of something later, please feel free to contact me.

Thank you for your time.
